# Comparative decision-making analysis of extracorporeal membrane oxygenation candidacy based on a survey of pediatric critical care fellow and attending physicians☆

**DOI:** 10.1051/ject/2025056

**Published:** 2026-06-19

**Authors:** Ish Sethi, Brenna C. McCabe, Ryan W. Morgan, Samuel Rosenblatt, Jessica C. Fowler, Wynne E. Morrison, Adam S. Himebauch

**Affiliations:** 1 Perelman School of Medicine at the University of Pennsylvania Philadelphia PA USA; 2 Division of Pediatric Critical Care and Hospital Medicine, Department of Pediatrics, Columbia University Irving Medical Center and Morgan Stanley Children’s Hospital New York NY USA; 3 Division of Critical Care Medicine, Department of Anesthesiology and Critical Care Medicine, Perelman School of Medicine at the University of Pennsylvania The Children’s Hospital of Philadelphia Philadelphia PA USA; 4 Department of Pediatrics, The Justin Michael Ingerman Center for Pediatric Palliative Care, The Children’s Hospital of Philadelphia Philadelphia PA USA; 5 ECMO Center, The Children’s Hospital of Philadelphia Philadelphia PA USA

**Keywords:** Extracorporeal membrane oxygenation, Critical care, Decision making, Pediatric intensive care units, Education, medical, graduate

## Abstract

*Background*: Extracorporeal membrane oxygenation (ECMO) candidacy decisions for children with respiratory failure can be variable among pediatric critical care attending physicians, and prior studies showed that baseline functional status and underlying neurological conditions influence this decision. However, there are limited data regarding factors influencing pediatric critical care fellows’ ECMO candidacy decisions and their alignment with attending physicians. This study aimed to identify patient characteristics influencing fellows’ ECMO candidacy decisions and measure concordance with attending decisions. *Methods*: This study was a planned secondary analysis of a prospective, single-center, cross-sectional study at a quaternary pediatric ECMO referral center. Pediatric critical care fellows and attending physicians caring for children admitted with acute respiratory failure were surveyed within 72 h of initiation or escalation of respiratory support. The primary exposure was patient functional status at admission, measured by the functional status score (FSS), and was categorized as Normal/Mild Dysfunction (FSS 6–9) or Moderate/Severe Dysfunction (FSS >10). Multivariate logistic regression clustered by fellow evaluated factors influencing ECMO candidacy assessments. Cohen’s kappa measured concordance between fellow and attending decisions. *Results*: Eighty surveys were completed by 21 pediatric critical care fellows. Fellows identified 19% of patients as ECMO non-candidates. After adjustment for age, moderate/severe admission dysfunction significantly reduced the odds of ECMO candidacy (aOR 0.11, 95% CI 0.03–0.51, *p* = 0.005). Overall, concordance between fellows and attendings was moderate (*κ* = 0.56), with junior fellows having minimal agreement (*κ* = −0.12). Fellows focused primarily on baseline functional status and comorbidities, while attendings considered additional factors, including long-term prognosis, organ failure irreversibility, and ECMO-related risks in candidacy assessments. *Conclusion*: Admission functional status influences pediatric critical care fellows’ ECMO candidacy decisions, with moderate concordance observed between fellows and attending physicians. The identified discrepancies emphasize the importance of structured education and targeted mentorship programs to enhance consistency in ECMO candidacy assessments, especially among junior trainees.

## Introduction

Extracorporeal membrane oxygenation (ECMO) is a therapeutic option for many patients experiencing severe or refractory acute respiratory failure [1, 2]. Despite its life-saving potential, ECMO is not universally applicable to all critically ill pediatric patients and is associated with significant risks and expenditure of resources [3, 4]. The decision to initiate ECMO involves numerous complex factors, highlighting the need for careful, individualized consideration [5]. While guidelines from the Extracorporeal Life Support Organization (ELSO) provide general indications and contraindications for ECMO, these guidelines are not prescriptive or comprehensive. The lack of standardized criteria and different resources leads to variability in ECMO candidacy decisions across institutions and among healthcare providers [6], which may lead to inequities in the application of this therapy [7].

Previous research has highlighted that baseline quality of life significantly influences decisions about ECMO candidacy for pediatric critical care attending physicians, and more than one third of pediatric ECMO patients have preexisting neurological disorders or neurofunctional differences [8, 9]. The Functional Status Score (FSS) is a validated pediatric measure that assesses functional status across six domains (mental status, sensory, communication, motor, feeding, and respiratory function) with higher scores indicating greater impairment and has been used in prior studies involving pediatric ECMO patients [9–14]. Further, ECMO candidacy decisions remain highly variable, with substantial provider-dependent differences in selection criteria. A recent study of adult ECMO decision-making found that even within a single institution, ECMO denial criteria were inconsistently applied, and 90% of candidates had at least one characteristic that was considered a prohibitive contraindication for another patient [15].

While prior research has explored decision-making variability at the institutional level and by attending physicians, it is unknown what pediatric critical care physician trainees view as important factors in considering ECMO candidacy and how this perception compares to that of attending physicians. Understanding the potential differences in ECMO candidacy decision-making across physician experience levels, and between multidisciplinary team members, is a crucial step in creating consistent decision-making guidelines to optimize patient- and family-centered outcomes.

The primary objective of this study was to determine the association between patient characteristics and pediatric critical care fellow physicians’ perception of ECMO candidacy for patients with acute respiratory failure. The secondary objective was to examine concordance in the decision-making between fellows and pediatric critical care attending physicians. This study utilized the FSS to quantify patient functional status at admission and hypothesized that: 1) worse patient admission FSS would be negatively associated with pediatric critical care fellow physicians’ assessment of ECMO candidacy, and 2) fellows and attendings would have high concordance when assessing candidacy.

## Methods

### Study design

This study was a planned, secondary analysis of a prospective, single-center, cross-sectional study conducted at the Children’s Hospital of Philadelphia (CHOP) Pediatric Intensive Care Unit (PICU). The Consensus-Based Checklist for Reporting of Survey Studies (CROSS) is reported in Supplementary Table 1.

### Subject eligibility

The primary study protocol has been previously published [9]. For this study, pediatric critical care medicine fellow physicians were eligible to participate if directly involved in the care of a qualifying patient at the time of initiation or escalation of ventilatory support. Patients were included in this study if they were less than 18 years of age on admission to the CHOP PICU, had acute respiratory failure requiring invasive mechanical ventilation, and were within 72 h of endotracheal intubation or, if tracheostomy was present, within 72 h of escalation from baseline respiratory support.

Participants were unaware of the primary hypothesis of this study [9]. An online Research Electronic Data Capture (REDCap, Vanderbilt University, Nashville, TN, USA)-based survey was sent to eligible physicians via email. The survey design, structure, and content are detailed in the primary study publication [9]. Survey items assessing which factors physicians considered most important in ECMO candidacy were developed by the study investigators. They were informed by prior literature on ECMO decision-making, but were not adapted from a standardized tool. The fellow physician involved with the study design and implementation (author initials B.C.M.) did not participate in the surveys.

### Data collection

While the patient’s medical history and co-morbid conditions were available at the time of survey participation, the functional status at PICU admission as measured by FSS was not [9]. Trained nurses who were independent of patient care abstracted data for FSS domains. For this analysis, FSS scores were divided into 2 categories: 1) Normal/Mild Dysfunction (Admission FSS 6–9); 2) Moderate/Severe Dysfunction (Admission FSS > 10). Additional patient data, such as Pediatric Index of Mortality 3 (PIM3) scores and comorbidities, were abstracted from the electronic health record and Virtual PICU Systems (VPS, Los Angeles, CA, USA) standardized data elements.

### Statistical analyses

Nonparametric summary and comparative statistics were used to analyze both patient and fellow physician information. Univariable and multivariate logistic regression analyses, clustered by individual pediatric critical care fellow, were performed for the primary outcome of ECMO candidacy. Given the number of patients not considered ECMO candidates, we used only the variables of patient age and admission FSS category in the multivariable logistic regression analyses to avoid overfitting the model. Age was specifically chosen as it may influence a clinician’s decision on ECMO candidacy when considering a patient’s future quality of life and recovery potential from acute critical illness.

We used Cohen’s Kappa to measure the concordance between the ECMO candidacy assessments for patients assessed by both fellow and attending physicians. For this study, concordance was defined as both the fellow and attending identifying the same patient as either an ECMO candidate or a non-candidate, whereas non-concordance was defined as a discrepancy between fellow and attending assessments for the same patient. All analyses were performed using R Statistical Software (R Foundation for Statistical Computing, Vienna, Austria).

## Results

### Characteristics of physicians and patients

Of 176 eligible patient encounters, 80 surveys were completed by pediatric critical care fellow physicians (response rate 45%). All patients had corresponding surveys completed by attending physicians. There were 21 individual fellow responders and 31 individual attending responders, with an average of 3.8 responses per fellow and 2.6 responses per attending; 18 fellows and 19 attendings participated in the survey more than once. More than 50% of attendings had >5 years of experience, while the median experience level in pediatric critical care for fellows was two years (Supplementary Table 2). The distribution of fellow respondents by training level was 38% [8] first-years, 33% [7] second years, and 29% [6] third years.

The median patient age was 3.5 years (IQR 1.1–9.0 years), and 60% of patients were male (n=48). The median admission FSS was 7 (IQR 6–12). The most common FSS category was normal/mild dysfunction (75%), with the moderate/severe dysfunction category comprising 25% (Table 1). Sixty-eight patients (85%) had at least one comorbidity, and the most common comorbidities were chronic respiratory failure (31%), developmental delay or disorder (31%), and epilepsy or history of seizures (23%) (Table 1).

**Table 1:** Patient demographics and characteristics comparison of non-ECMO and ECMO candidates as assessed by pediatric critical care fellow physicians.

Variable	Total (*N* = 80)	Non-ECMO candidates (*N* = 15)	ECMO-candidates (*N* = 65)	*p*-value
Age (years), median (IQR)	3.5 (1.1–9.0)	7 (4.5–14.0)	2.9 (0.9–8.0)	0.008
Sex, *n* (%)				0.14
Male	48 (60%)	12 (80%)	36 (55%)	
Female	32 (40%)	3 (20%)	29 (45%)	
Race, *n* (%)				0.62
American Indian or Alaska Native	1 (1.3%)	0 (0%)	1 (2%)	
Asian	3 (3.8%)	1 (7%)	2 (3%)	
Black or African American	16 (20%)	5 (33%)	11 (17%)	
Other/mixed	14 (17.5%)	2 (13%)	12 (18%)	
Unspecified	4 (5%)	0 (0%)	4 (7%)	
White	42 (52.5%)	7 (47%)	35 (54%)	
Admission FSS score, median (IQR)	7 (6–12.3)	16 (11–21)	6 (6–23)	0.002
Admission FSS category, *n* (%)				0.003
Normal to mild dysfunction	55 (68.7%)	5 (33.3%)	50 (76.9%)	
Moderate dysfunction	25 (31.3%)	10 (66.7%)	15 (23.1%)	
Comorbidities, *n* (%)				
Previously healthy	12 (15%)	1 (7%)	11 (17%)	
Prematurity	16 (20%)	3 (20%)	13 (20%)	
Pulmonary hypertension	14 (17.5%)	2 (13%)	12 (18%)	
Chronic lung Disease	14 (17.5%)	3 (20%)	11 (17%)	
Hematologic disease, non-oncologic	4 (5%)	3 (20%)	1 (2%)	
Oncologic diagnosis, solid	9 (11.3%)	3 (20%)	6 (9%)	
Oncologic diagnosis, liquid	4 (5%)	0 (0%)	4 (6%)	
SCT or BMT transplant recipient	1 (1.3%)	0 (0%)	1 (2%)	
Solid organ transplant recipient	0 (0%)	0 (0%)	0 (0%)	
Kidney disease (acute or chronic)	12 (15%)	5 (33%)	7 (10.8%)	
Epilepsy or history of seizure	18 (22.5%)	6 (40%)	12 (18%)	
Cerebral palsy	6 (7.5%)	1 (7%)	5 (8%)	
Developmental delay or disorder	25 (31.3%)	7 (47%)	18 (27.8%)	
HIE	4 (5%)	2 (13%)	2 (3%)	
Neuromuscular disorder	6 (7.5%)	1 (7%)	5 (8%)	
Thoracic insufficiency syndrome or neuromuscular scoliosis	4 (5%)	0 (0%)	4 (6%)	
Genetic syndrome	16 (20%)	2 (13%)	14 (22%)	
Chronic static encephalopathy	2 (2.5%)	0 (0%)	2 (3%)	
Hydrocephalus	8 (10%)	3 (20%)	5 (8%)	
Endocrinopathy	16 (20%)	9 (60%)	7 (11%)	
Chronic respiratory failure	25 (31.3%)	8 (53%)	17 (26%)	
Asthma or reactive airway disease	10 (12.5%)	3 (20%)	7 (11%)	
Cystic fibrosis	1 (1.3%)	0 (0%)	1 (2%)	
History of ECMO	1 (1.3%)	0 (0%)	1 (2%)	
History of cardiac arrest	3 (3.8%)	2 (13%)	1 (2%)	
Cardiac disease or dysfunction	17 (21.3%)	4 (27%)	13 (20%)	
Chronic feeding intolerance or intestinal failure	6 (7.5%)	2 (13%)	4 (6%)	

Abbreviations: BMT: bone marrow transplant; ECMO: extracorporeal membrane oxygenation; FSS: functional status score; HIE: hypoxic-ischemic encephalopathy; IQR: interquartile range; PICU: pediatric intensive care unit; PIM3: Pediatric Index of Mortality; PRISM: Pediatric Risk of Mortality; SCT: stem cell transplant.

### Patient characteristics associated with ECMO candidacy

Of the 80 patients who had fellow survey responses, 65 (81%) were perceived to be ECMO candidates, and 15 (19%) were not perceived to be ECMO candidates. These patients differed from each other in age and admission FSS category (Table 1).

In univariate logistic regression analysis, older patient age and higher admission PIM3 scores were associated with lower odds of perceived ECMO candidacy (OR 0.89 and 0.61, [*p* = 0.003] and [*p* = 0.02], respectively). An admission FSS category of moderate/severe dysfunction (OR 0.09, [*p* = 0.001]), and comorbidities including endocrinopathy (OR 0.08, [*p* = 0.0001]), kidney disease (OR 0.24, [*p* = 0.03]), and chronic respiratory failure (OR 0.31, [*p* = 0.003]) were also associated with lower odds of perceived ECMO candidacy (Supplementary Table 3). After adjusting for age, multivariate logistic regression demonstrated that patients with admission FSS category of moderate/severe dysfunction had lower odds of being considered an ECMO candidate compared to patients who had an admission FSS category of normal/mild dysfunction (aOR 0.11, 95% CI 0.03–0.51 [*p* = 0.005]).

### Concordance between fellows and attendings

**Figure 1:**
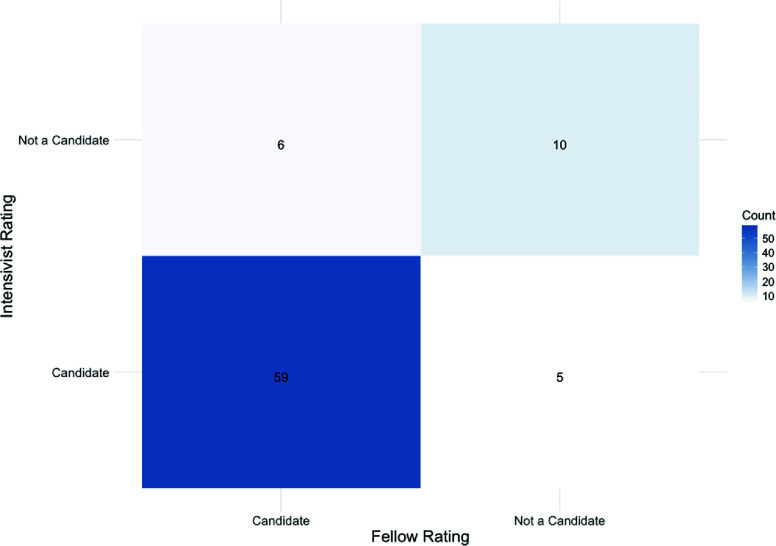
Concordance between pediatric critical care fellow and attending physician assessment of ECMO candidacy.

Fellows identified 15 patients (19%) as non-candidates for ECMO, while attendings identified 16 patients (20%) as non-candidates. Overall, there was a moderate level of agreement between the fellows and attendings (*κ* = 0.56, Figure 1). Specifically, 5/15 (33%) patients considered non-candidates by fellows were considered candidates by attendings, and 6/16 (38%) patients considered non-candidates by attendings were considered candidates by fellows. When comparing concordance between attendings and fellows by years of experience, senior (2^nd^-year and 3^rd^-year) fellows had high agreement (*κ* = 0.72) with attendings, while junior (1st-year) fellows showed little to no agreement (*κ* = −0.12). Junior fellows reported an average confidence score of 2.80/4, senior fellows reported an average confidence score of 3.10/4.00, with fellows overall reporting an average confidence score of 3.01/4.00 vs 3.29/4.00 amongst attendings [*p* = 0.1].

Patients for whom fellows and attendings disagreed on ECMO candidacy had worse admission functional status (median admission FSS 14 [IQR 10–21]) compared to those for whom fellows and attendings agreed (median admission FSS 6 [IQR 6–11], *p* = 0.024). There were no differences in patient age or severity of illness as measured by PIM 3 scores between agreement and disagreement cases.

### ECMO candidacy factors considered by fellow and attending physicians

**Figure 2:**
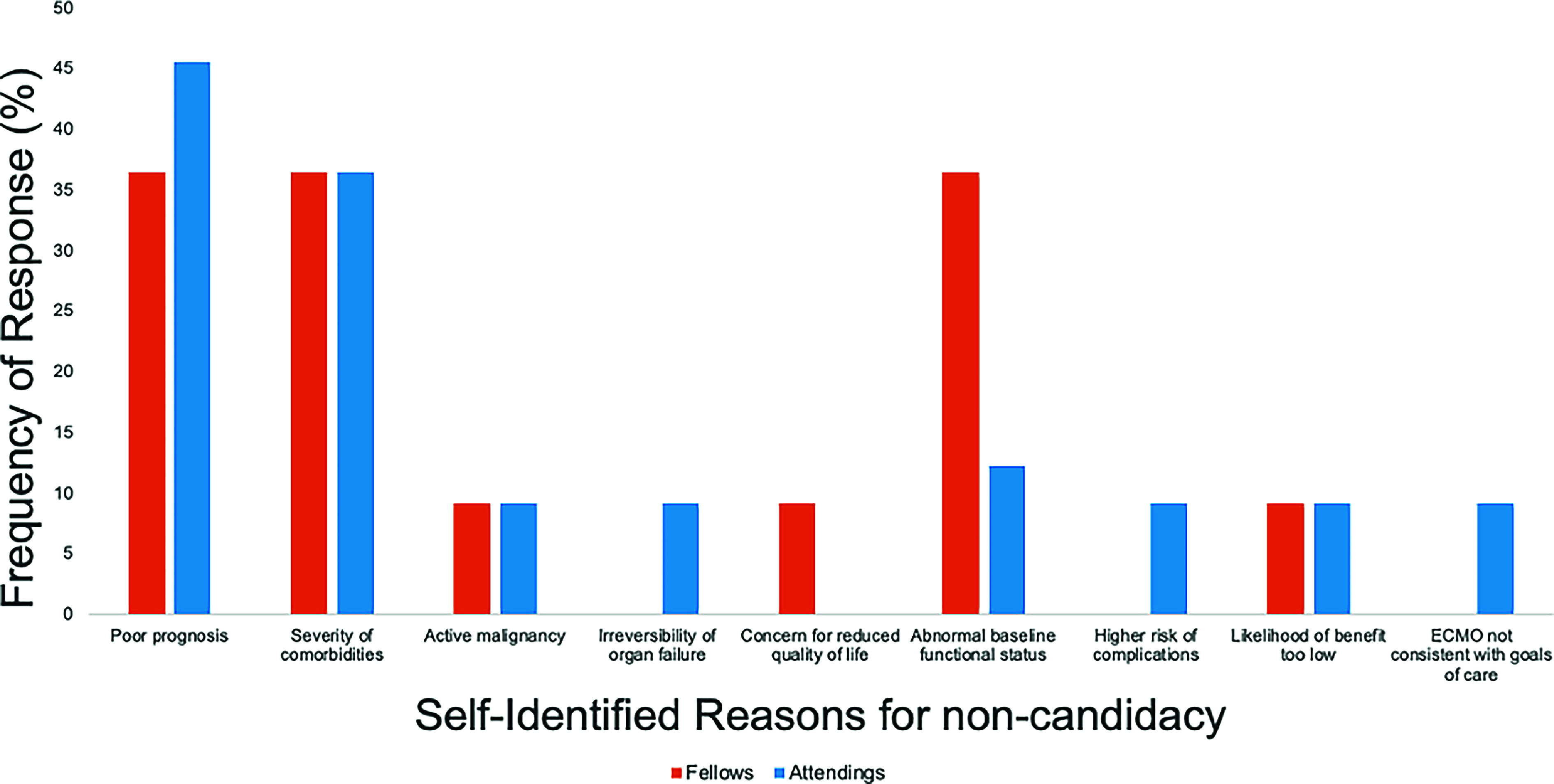
Frequency comparison between fellow-attending dyads of self-identified reasons for patient non-candidacy for ECMO (*N* = 80).

Fellows most frequently cited poor prognosis of a chronic illness or genetic condition (36.4%), severity of pre-existing comorbidities (36.4%), and abnormal baseline functional status (36.4%) as reasons for not considering a patient an ECMO candidate (Figure 2). Similarly, attendings identified poor prognosis of a chronic illness or genetic condition (45.5%) and severity of pre-existing comorbidities (36.4%) as key factors in their decisions. However, attendings also incorporated additional, potentially more subjective, nuanced factors, such as active malignancy (9.1%), irreversibility of organ failure (9.1%), and a higher-than-average risk of ECMO-related complications (9.1%).

## Discussion

Patient admission FSS category of moderate or severe dysfunction was associated with decreased odds of being perceived as an ECMO candidate by pediatric critical care fellows in this secondary analysis of a prospective, cross-sectional study. There was only moderate concordance between fellows and attendings, with senior fellows having the highest concordance and junior fellows having the lowest concordance. Approximately one-third of patients were differentially considered candidates or non-candidates between attendings and fellows. Patients who were younger and those with less severe functional impairment were more likely to be considered ECMO candidates for both fellows and attendings, but self-reported factors that influenced ECMO candidacy decisions differed between the physician groups.

Prior research has consistently emphasized that baseline functional status and pre-existing neurological conditions are pivotal factors in ECMO candidacy decisions, and trained providers often weigh their perceptions of a patient’s pre-illness quality of life heavily when deciding whether to recommend ECMO, especially in pediatric populations [8–11]. Our study is the first to extend this understanding to pediatric critical care trainees and suggests that, while there is an expected progression of learning among trainees to generally align assessments of ECMO candidacy with experienced attendings, discrepancies may arise in more complex cases involving older children or for patients with significant comorbidities. Attendings prioritized poor prognosis, irreversible organ failure, high complication risks, and alignment with goals of care more than fellows when assessing ECMO candidacy. These considerations may reflect more nuanced elements of decision-making that fellows have less experience incorporating early in their training. Attendings, with greater clinical experience, may be more attuned to these broader dimensions of risk–benefit assessment and patient- and family-centered care.

Prior studies have shown substantial differences in ECMO candidacy decisions between experienced ECMO program directors at different institutions and adult clinicians at the same institution [15, 16]. Similarly, our study demonstrated variability in ECMO candidacy decisions between fellows and attendings in a single-center study without variability in institutional culture or resource availability. The variability in candidacy decisions between fellows and attendings highlights the opportunity for educational initiatives to ensure consistency of ECMO candidacy assessments both within and across institutions. Training programs should focus on equipping fellows to make informed decisions, ideally in a patient- and family-centered manner with inclusion of patients (as able), families, and multidisciplinary medical and surgical teams within the context of their institutional resources and environment. Future research should focus on developing targeted educational interventions, such as simulation-based training and mentorship, to enhance fellows’ decision-making and communication skills to improve consistency in ECMO candidacy assessments. This education may be particularly applicable to training programs that do not have a high ECMO patient volume. Additionally, exploring the influence of institutional factors on decision-making could help create adaptable and more standardized guidelines.

We observed only moderate concordance between fellow and attending assessments of ECMO candidacy in this study, with junior fellows having minimal concordance. Although this is a widely acknowledged reality in training programs and intuitively makes sense, our study quantifies differences between junior and senior trainees. During the timeframe of this study, most of the first-year fellow responders were in their first 6 months of fellowship. As expected, concordance did increase between attendings and senior fellows. Furthermore, fellows prioritized baseline functional status and pre-existing comorbidities, while attendings incorporated additional considerations such as long-term prognosis and ECMO-specific risks. This mirrors the finding from Rubin et al. that providers interpret ECMO contraindications differently, even when faced with similar cases [15]. Longitudinal studies following trainees throughout their fellowship and early post-fellowship practice could provide valuable insights into how decision-making evolves with the transition to unsupervised practice.

In our cohort, comorbidities such as endocrinopathies, kidney disease, and malignancy were associated with lower odds of ECMO candidacy. It is important to note that these categorizations captured a broad spectrum of conditions, ranging from relatively isolated diagnoses to multifactorial disease states with significant multisystem involvement. In particular, the association between endocrinopathy and lower perceived ECMO candidacy likely reflects this heterogeneity and may be driven by more complex cases rather than isolated endocrine diagnoses. Additionally, while pediatric surgeons, oncologists, nephrologists, and endocrinologists play a critical role in the multidisciplinary management of critically ill children, their perspectives were not directly captured in our survey, nor were we able to quantify the influence of any clinically related discussions with specialists on the critical care physician assessments of ECMO candidacy. Future studies should incorporate the input of subspecialist consultants, as their expertise may substantially influence fellows’ and attendings’ ECMO candidacy decisions.

Another important consideration for ECMO candidacy is the use of physiological data and specific clinical cut-points to help identify patients at the highest mortality risk. Prior work demonstrated that physiologic thresholds in neonatal and pediatric respiratory and cardiac patients can be used to define late ECMO implementation and predict increased mortality risk [16, 17]. Although our study focused on physician perceptions rather than physiologic parameters, it is plausible that, given the physicians were directly involved in the care of these patients, some physiologic data were pertinent to their candidacy decisions. Future research could integrate physiologic criteria with provider assessments to develop more standardized and evidence-based candidacy frameworks.

Our study has several limitations. Our relatively small sample size and single-center design may limit the generalizability of our findings to other institutions and may not reflect geographic or institutional variability. Further, due to the feasibility and focus of the research questions, critical care advanced practice providers, perfusionists, ECMO specialists, non-PICU pediatric subspecialists, or parents were not included in this survey. There is a potential for sampling bias as there were variable amounts of repeated participation for both fellow and attending physicians, which we attempted to mitigate by clustering the logistic regression by fellow. The response rate for the survey from fellows was 45%, and it is possible that response bias could result if fellows were less likely to respond to a survey if they considered their patient a non-candidate or more likely to respond if they had particularly strong inclinations regarding candidacy. Another limitation is the potential for ‘group think’ bias, as fellows may adapt their decision-making over time to align with attending physicians’ preferences. This dynamic is particularly plausible in a single-center model where the same group of attendings supervises all trainees. While this effect cannot be directly measured in our study, it may contribute to increasing concordance observed among senior fellows.

We did not measure or survey parental or family considerations of quality-of-life assessments, which may or may not correlate with FSS scores. Family perspectives on quality-of-life are important considerations in the discussion of ECMO candidacy for any pediatric patient. Additionally, all patients in this study were cared for in a dedicated PICU, and none were admitted to a cardiac ICU. As such, our results may not be generalizable to centers with mixed units or to patients with congenital or acquired cardiac disease for whom cardiologists, congenital cardiothoracic surgeons, and cardiac intensivists play a prominent role in ECMO candidacy decisions. Finally, while our study did ask opinions about actual patients, the consideration of ECMO was hypothetical and may have biased some fellow and attending responses.

In conclusion, patient functional status on admission and age are significant factors that pediatric critical care fellows consider when assessing ECMO candidacy for pediatric patients with severe acute respiratory failure. Although agreement with pediatric critical care attendings did increase throughout training, overall, there was only moderate concordance between fellows and attendings, and there were differences in the factors that attendings prioritize compared to fellows when making ECMO candidacy decisions. These findings highlight the need to better understand how decision-making evolves with experience and to better standardize and optimize decision-making processes of the multidisciplinary critical care team and families.

## Abbreviation

BPD: bronchopulmonary dysplasia
FSS: functional status score
IVH: intraventricular hemorrhage
PIM3: Pediatric Index of Mortality
Ref: Reference

## Data Availability

The data set used for this manuscript is available upon reasonable request to the corresponding author (ASH).

## Supplementary material

*Supplementary Table 1*: Checklist for Reporting Of Survey Studies (CROSS). *Supplementary Table 2*: Characteristics of pediatric critical care fellow and attending physician study participants. *Supplementary Table 3*: Univariable logistic regressions for perceived ECMO candidacy by critical care fellow physicians. Supplementary materials for this article are available at https://ject.edpsciences.org/10.1051/ject/2025056/olm.
